# A structural causal model for robot-assisted upper-limb neurorehabilitation

**DOI:** 10.3389/fresc.2026.1819379

**Published:** 2026-07-14

**Authors:** Sivakumar Balasubramanian

**Affiliations:** 1 Department of Bioengineering, Christian Medical College Vellore, Vellore, Tamil Nadu, India; 2 School for Health and Rehabilitation Sciences, The University of Queensland, Brisbane, Queensland, Australia

**Keywords:** directed acyclic graph, mechanistic modeling, precision neurorehabilitation, robot-assisted therapy, stroke, structural causal model, upper-limb rehabilitation

## Abstract

Upper-limb robot-assisted neurorehabilitation in stroke yields modest improvements in impairments that do not generalize to functional outcomes, with substantial variability across patients. In response, there is increasing interest in precision neurorehabilitation through mechanistically driven, tailored robot-assisted therapy for individual patients. Such approaches require models that support interventional reasoning about therapy parameters (e.g., “what if we increase robotic assistance or dose for this patient?”), rather than providing purely associational findings such as biomarkers correlated with recovery. Leveraging recent developments in causal inference, this paper presents a structural causal model of robot-assisted therapy for the upper limb in the form of a directed acyclic graph. The graph encodes key constructs identified in the robot-assisted neurorehabilitation literature as nodes and represents their known or hypothesized causal influences as directed edges, reflecting current domain knowledge. We describe the components of the causal graph in detail and show how it can account for several observed phenomena in robot-assisted therapy, while also yielding testable predictions in the form of interventional effects. We then highlight important limitations of the proposed causal model, before presenting a conceptual example of how a fully specified causal graph could help answer questions about attainable outcomes and optimal therapy parameters for individual patients. The proposed concrete causal graph must be empirically investigated to test its validity and refine its causal structure through observational and experimental studies. We anticipate that this proposed causal graph will serve a catalytic role in advancing our mechanistic understanding of robot-assisted therapy, which may hold the key toward improving individual patient outcomes with robot-assisted therapy.

## 1 Introduction

“Problems are inevitable. Problems are soluble.”

—David Deutsch, *The Beginning of Infinity*

The last three decades have seen a tremendous activity in the development and clinical evaluation of a wide range of robotic devices (1) for upper-limb (UL)^1^ therapy following stroke. Although most current UL rehabilitation robots are complex, expensive, and mostly restricted to the clinic, there is an increasing trend towards simpler, portable, and affordable devices designed to work across the continuum of care (2–6). These devices present new opportunities for increasing therapy dose beyond what is possible with conventional therapy (5).

Existing clinical evidence for UL robot-assisted therapy (RAT) in stroke indicates that robots can safely provide high intensity therapy; they can support hundreds of movements in a session (7). A recent meta-analysis by de Iaco et al., which included 90 randomized controlled trials (≈2,500 subjects), found that UL RAT shows a small improvement of ≈ 2 points on the UL Fugl-Meyer Assessment (FMA) scale (8). However, these small improvements in motor impairments do not generalize to UL capacity (8), i.e., the ability to lift/carry objects, fine hand function, etc. De Iaco et al. (8) also observed substantial response heterogeneity in the UL FMA scores in their meta-analysis [supplementary figure with the forest plot in (8)]; response heterogeneity is a common problem in medical treatments (9, 10). The recent call for precision neurorehabilitation (11–14) is an effort to minimize this response heterogeneity through tailored interventions and maximize individual treatment responses. De Iaco et al. (8) concluded their meta-analysis with a recommendation for mechanistically-driven development of future RAT. A mechanistic model of RAT can provide the scaffold to build precision neurorehabilitation approaches. Such a model has been called by different terms in the current literature, such as computational neurorehabilitation model (13), digital twin (12), or a causal model (11).

Previous studies have identified biomarkers of spontaneous or therapy-driven post-stroke recovery (14–19). The PREP2 algorithm uses the shoulder-abduction-finger-extension (SAFE) score along with motor evoked potential (MEP) measurements or brain imaging data, early after stroke, to predict the amount of UL function at 6 months post-stroke (20, 21). The presence or absence of MEP has been found to be a good predictor of the amount of UL impairments as measured by the FMA (22). The extent of injury to the motor tracts measured through brain imagining predicted the sensorimotor gains following robot-assisted hand therapy (18). Another study found that the somatosensory integrity of the paretic hand predicted therapy-driven gains in robot-assisted finger therapy (16). A recent three-arm randomized controlled trial found that proprioceptive status of the fingers can predict responsiveness to robotic and non-robotic therapy (15). These are examples of associative models that identify specific factors or biomarkers correlated with recovery, which can be used for patient stratification, as was suggested by Farrens et al. (15). Although, these studies are important steps towards a deeper understanding of RAT and developing precision neurorehabilitation strategies, further work is needed to build a comprehensive mechanistic model of RAT that can be exploited for optimal therapy prescription.

Frameworks for such models exist in the current literature. Reinkensmeyer et al. (13) proposed the idea of *computational neurorehabilitation* models that are detailed, mechanistic dynamical models typically in the form of differential equations modeling the neural plasticity and sensorimotor recovery processes employing physiologically meaningful latent and observable states. Liew et al. (12) proposed the idea of *digital twins* for neurorehabilitation, which are virtual replicas of the patient and their recovery process that can be used to simulate and optimize therapy. They advocate the use of *collaborative AI* for this purpose. Cotton et al. (11) proposed the use of *causal models* to understand the mechanisms underlying recovery and to guide therapy decisions. These three frameworks share the common goal of capturing the causal, mechanistic process underlying sensorimotor recovery, albeit through different approaches and varying levels of detail. They also differ from the associational models discussed before in that they enable interventional reasoning, i.e., what will happen if we administer an intervention or change a therapy parameter.

Taking heed of the growing call for theory-driven approaches in rehabilitation (8, 11, 13, 23, 24), and drawing on causal inference methods (25–27), this paper proposes a structural causal model (SCM) for RAT for UL stroke neurorehabilitation. The model synthesizes current evidence for RAT-driven sensorimotor recovery, explicitly stating its mechanistic assumptions and hypotheses. We first present a general computational neurorehabilitation-type model of sensorimotor recovery, from which a simplified RAT-specific SCM is derived. We then provide a detailed description of the proposed model, which includes the definition of the SCM’s components and their hypothesized causal links. Following this, we discuss the implications of this proposed model, and its current limitations. We then briefly demonstrate how RAT can be tailored to individual patients, once a fully specified SCM for RAT is available. Finally, we conclude with a brief illustration of how the parameters of the proposed SCM can be learned from data through appropriately designed experimental and observational studies.

## 2 A general dynamic model of sensorimotor recovery in neurorehabilitation

“Imagine how much harder physics would be if electrons had feelings.”

—Richard Feynman

Motor recovery is a complex, continuous-time dynamic process affected by various subject-specific, environmental, and therapy-related factors. The knowledge of the underlying laws governing this process would empower clinicians to maximize recovery for individual patients through personalized therapy. A computational neurorehabilitation (13) model can capture this process through a mechanistic state-space vector differential equation, given as follows,

$$\begin{array}{cc} \dot{x}\left(t\right) & =f\left(x\left(t\right),u\left(t\right);p\right)\\ y\left(t\right) & =g\left(x\left(t\right);p\right)+\epsilon \left(t\right) \end{array}$$ (1)

where, x is the set of all dynamic factors relevant to the recovery process that may interact with interventions or other subject-specific traits. The state vector could include factors spanning the physiological, biomechanical, and psychological domains. Some potential components of the state vector x include cortical neuronal excitability (28), resting state brain connectivity (29, 30), sensory acuity (30, 31), motor coordination (32), and intrinsic motivation (33). On the other hand, the parameter vector p constitutes subject-specific (quasi-) stable traits, that can moderate the interaction between the state variables and the therapeutic intervention u. This includes factors such as age (34), cognitive reserve (35, 36), lesion characteristics (37–39), residual corticospinal tract integrity (20–22), personality (40), caregiver/family support (41), and socioeconomic status (42, 43). The vector u is the list of intervention variables under the clinician’s control. These include prescribed dose and intensity, task complexity, therapy modality, and feedback modality and schedule. The function f captures how state variables (x), the intervention variables (u), and the parameter vector (p) interact and influence the temporal evolution of the state $\left(\dot{x}\right)$. The state vector is often not directly measurable, but influences the measurable variables represented by the measurement vector y. The measurement vector y could include standard clinical assessments (e.g., Fugl-Meyer assessment, action research arm test), quantitative sensorimotor assessments (e.g., range of motion, muscle strength, movement smoothness), UL functioning (44), patient reported outcomes (e.g., motor activity log), and neurophysiological measures (e.g., EMG, motor evoked potentials, and resting state EEG). The relationship between the measurement vector y and the state vector x is captured by the function g. The vector ϵ represents output measurement noise, along with unmodeled dynamics affecting state evolution.

Using the dynamic model in Equation 1, choosing an optimal therapy program or policy to efficiently maximize recovery can be posed as the following constrained optimization problem,

$$\begin{array}{cc} u^{*}\left(t\right)=\underset{u(t)}{\arg\max} & \;J(y,u;p)\\ \text{subject to} & \;\dot{x}=f(x,u;p)\\ & \;y=g(x)+\epsilon \\ & \;c\left(x,u,p\right)\leq 0 \end{array}$$ (2)

where, J is a cost function representing the therapy goals (e.g., maximize sensorimotor recovery, while minimizing therapy time, effort, and cost). The function c captures the set of general and subject-specific constraints (e.g., maximum cost affordable by the subject, constraints on daily therapy time and intensity, etc.). One could, in principle, solve this problem in Equation 2, using tools from optimal control theory employing numerical approaches (45), provided x, p, u, and y are fully specified, the complete knowledge of f and g and the nature of ϵ are available.

Isolated models of selected sensorimotor learning processes exist for simple laboratory based movements, for example, sensorimotor adaptation (46), use-dependent learning (47), etc. However, currently no mechanistic models of sensorimotor learning or recovery exist that can be exploited for neurorehabilitation. Comprehensive models integrating different learning processes (48–50) and general contexts (51), accounting for subject-specific co-variates, and therapy parameters, are lacking. We also are unlikely to have such a detailed mechanistic model with high temporal resolution in the near future. However, the model in Equation 1 serves as a principled conceptual framework to formalize and reason about the general recovery process in neurorehabilitation, even though this may be unoperationalizable in practice.

## 3 Structural causal model of robot-assisted therapy

Our current knowledge and understanding of the relevant factors in neurorehabilitation and their interactions is incomplete and uncertain. We know only a few of the relevant states x and parameters p of Equation 1 with varying levels of certainty. The interactions (f,g) between these variables are either only sparsely supported by direct experimental evidence in neurorehabilitation or are inferred from evidence from related fields, such as motor/skill learning. Though this partial knowledge does not allow us to build a model like Equation 1, it does naturally allow us to build structural causal models (SCM) (52). An SCM represents the key factors involved in a process, captures their known and hypothesized causal interactions, which can be learned from data and experiments, for interventional reasoning. The SCM can be thought of as a simpler, tractable approximation to coupled non-linear differential equations (53), such as Equation 1, for capturing causal relationships that can predict the effects of interventions.

**Figure 1:**
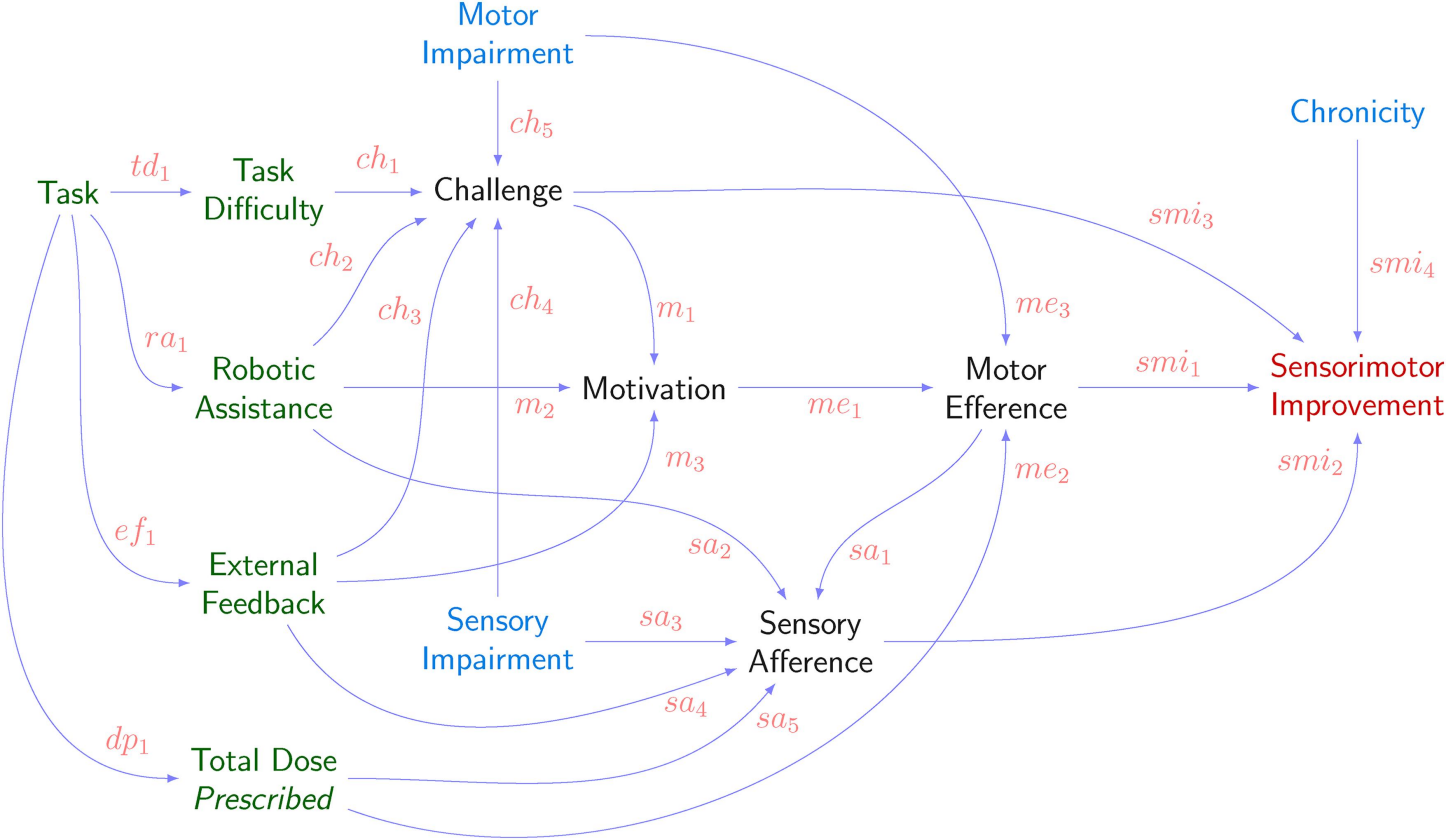
A directed acyclic graph representing the structural causal model of robot-assisted therapy. The colored nodes represent the type of node they are: red for target, green for ingredients, gray for mechanism nodes, and blue for subject-specific traits. The directed edges represent the known or hypothesized causal relationships between these nodes.

A directed acyclic graph (DAG) representing the proposed SCM for RAT is shown in Figure 1, with its relevant variables (as nodes), along with their causal relationships (directed edges). We will refer to this model as the RAT-SCM in the rest of the paper. When examining the SCM presented in Figure 1, several fundamental questions emerge: How were these nodes and edges chosen? What phenomenon does this model capture? Where is it applicable? What do the different nodes of the DAG represent? What are the mechanistic processes associated with the hypothesized causal links connecting these nodes? We will address these questions in the following subsections.

### 3.1 Choice of RAT-SCM nodes and edges

Building causal DAGs means choosing its nodes and directed edges. This choice is firmly grounded in domain knowledge, and guided by practical concerns such as model faithfulness, identifiability, and clinical utility. The RAT-SCM nodes were chosen to capture the tripartite structure of *treatment theory* (23, 54), which separates rehabilitation treatments into three components (23): (a) *target*—the specific aspect of a subject’s behavior or function that is intended for change through the treatment; (b) the treatment *ingredients* that are administered to change the target of interest; and (c) the *mechanism of action* through which the treatment ingredients change the intended target; these are known or hypothesized causal connections between the ingredients and the target(s). A summary of the categorization of the RAT-SCM nodes based on this structure is provided in Table 1, along with their connection to the computational neurorehabilitation model in Equation 1. The red-colored node in Figure 1 represents the treatment target—the relevant outcome measure for the RAT intervention. The five green-colored nodes in Figure 1 denote the ingredients of RAT, specifically the robot-specific parameters that capture the nature of training tasks, their difficulty, amount of robotic assistance, augmented sensory feedback during training, and the total prescribed therapy dose. The blue- and gray-colored nodes in Figure 1 are factors associated with the mechanism of action of RAT. The three blue-colored nodes (baseline sensorimotor impairments and chronicity) are patient-specific traits that can moderate the recovery process. The four gray-colored nodes represent constructs either supported or hypothesized for their role in the sensorimotor learning/recovery process. The restriction to three blue- and four gray-colored nodes is a deliberate choice to balance between model completeness, complexity, and the availability of empirical evidence for mechanistic interaction between the RAT-SCM nodes. In treatment theory, causality flows from the ingredients to the target, which was one of the principles guiding the choice of direction of the RAT-SCM edges. The choice of edges between two specific nodes was based on either direct or indirect empirical evidence, and logical consistency.

**Table 1:** Connection between the tripartite structure of treatments, the RAT-SCM (Figure 1), and the detailed general recovery model (Equation 1).

Treatment theory	Robot-assisted therapy SCM	General recovery model
Target	Sensorimotor Improvement	y
Ingredient	Task, Task Difficulty, External Feedback, Dose Prescribed, Robotic Assistance	u
	Directed edges between the different nodes	f(x,u;p) g(x;p)
Mechanism of action	Challenge, Motivation, Motor Efference, Sensory Afference	x
	Motor Impairment, Sensory Impairment, Chronicity	p

The nodes of the SCM are highlighted in italic in the second column.

The exact nature of the chosen RAT-SCM nodes and edges, along with some supporting evidence from the literature, are discussed in depth in Section 3.3. The literature in fields like neurorehabilitation and motor control/learning is complex and scattered. Thus, model development in these fields relies on literature synthesis—which can never be comprehensive—along with creative gap-filling guided by model consistency, elegance, and parsimony. As a result, the choices made in the proposed RAT-SCM are best treated as falsifiable hypotheses rather than as statements of fact about the sensorimotor recovery process. A summary of the chosen nodes and edges of the RAT-SCM is provided in Tables 2 and 3, respectively. Table 2 lists all the nodes, their types (defined in the table caption), and the potential measures/assessment tools for quantifying them. Table 3 lists all the causal edges in the RAT-SCM, indicating the rationale behind their existence. For each edge, the table indicates whether its inclusion is grounded in: (a) the definitions of the RAT-SCM nodes; (b) first principles; (c) empirical evidence; or (d) a hypothesis that remains to be verified experimentally.

**Table 2:** Operational summary of the RAT-SCM nodes.

Node	Type	Candidate measures/assessment tools
Task ($X_{T}$)	Ingredient	Presence or absence of task type. It is robot dependent.
Task Difficulty ($X_{TD}$)	Ingredient	A measure must include a combination of target distance, size, duration, etc. [e.g Fitts law (55)].
Robotic Assistance ($X_{RA}$)	Ingredient	Inverse dynamics based estimation through a model of the human-robot interaction; slacking metric (56).
External Feedback ($X_{EF}$)	Ingredient	Requires multivariate specification containing feedback modality (categorical), content, schedule, etc.
Total Dose Prescribed ($X_{TDP}$)	Ingredient	Prescribed active therapy time.
Chronicity ($X_{C}$)	Trait	Clinical record (onset date), e.g., acute/subacute/chronic for stroke.
Motor Impairment ($X_{MI}$)	Trait	FMA, joint ROM, strength, robotic assessments (57, 58)
Sensory Impairment ($X_{SI}$)	Trait	Monofilament, proprioception scales, robotic assessment (59, 60)
Challenge ($X_{Ch}$)	Latent	Success rate, task error, NASA-TLX (61), salivary α-amylase, etc.
Motivation ($X_{M}$)	Latent	Intrinsic motivation index (IMI) (33), stroke rehabilitation motivation scale (62), VAS 0–10, task adherence, etc.
Motor Efference ($X_{ME}$)	Latent	Training time (dose component); movement vigor, skin conductance, EEG error potentials, etc.
Sensory Afference ($X_{SA}$)	Latent*	No direct proxy. Must take into account the motor output, external feedback, and total therapy time.
Sensorimotor Improvement ($X_{SMI}$)	Outcome	ΔFMA, range of motion, movement speed, smoothness, accuracy, grip strength, finger coordination, etc. (8, 63)

Each node is classified by *type*—*Ingredient* (set by the therapy prescription), *Trait* (quasi-static observable variables related to the patient), *Latent* (a mechanistic mediator requiring indirect estimation; shaded rows), or *Outcome* (observable sensorimotor target of interest); Latent* indicates that this is a hypothesized node. The last column lists candidate measures/assessment tools, since several constructs do not yet admit a standard scale.

**Table 3:** Epistemic basis of the causal edges of the RAT-SCM.

Edge type	Edges (with key reference(s))
**Definitional**: the parent parametrises the child by construction	∙ $X_{T}\to X_{TD},X_{EF},X_{RA},X_{TDP}$
	∙ $X_{TDP}\to X_{ME},X_{SA}$: More prescribed time means more movement and more sensory input.
**First-principle**: follows necessarily from physical or physiological reasoning	∙ $X_{TD},X_{EF},X_{RA},X_{MI},X_{SI}\to X_{Ch}$
	∙ $X_{ME}\to X_{SA}$: More movement generates more sensory input.
	∙ $X_{SI}\to X_{SA}$: More sensory impairment implies poorer sensory input.
	∙ $X_{MI}\to X_{ME}$: More motor impairment implies poorer movements.
**Empirical**: supported by experimental or observational evidence	∙ $X_{Ch}\to X_{M}$ (67–69)
	∙ $X_{RA}\to X_{M}$ (19, 33)
	∙ $X_{EF}\to X_{M}$ (70–72)
	∙ $X_{M}\to X_{ME}$ (73–75)
	∙ $X_{EF}\to X_{SA}$ (76–78)
	∙ $X_{ME}\to X_{SMI}$ (49, 79, 80)
	∙ $X_{C}\to X_{SMI}$ (81–83)
**Hypothesized**: plausible but not yet directly tested	∙ $X_{Ch}\to X_{SMI}$: Challenge moderates the effect of voluntary movements and their sensory consequences on sensorimotor improvement.
	∙ $X_{RA}\to X_{SA}$: Robotic assistance has a parallel influence on sensorimotor improvement mediated by sensory afference, in addition to the one mediated by motivation.
	∙ $X_{SA}\to X_{SMI}$: Some evidence from the electrical stimulation literature (77) exists, but this evidence is indirect.

Edges are grouped by their primary basis, defined in the first column. Node symbols follow the notation of Section 3.3.

### 3.2 The phenomenon modeled by the RAT-SCM

“All models are wrong, but some are useful.”

—George E. P. Box

Two key properties should be noted to understand the scope of the proposed RAT-SCM. First, it represents a **forward pass of the general recovery model** (Equation 1) over a **fixed time interval** with **fixed therapy parameters**. And second, although the overall model represents the general RAT-driven recovery process, some of the model parameters are **robot-specific**. We elaborate on these properties below.

While Equation 1 describes the recovery process with infinite temporal resolution, the RAT-SCM in Figure 1 captures the same process over a fixed time interval, referred to as the *training epoch* T. The training epoch could correspond to a single therapy session (e.g., 30 min), a few therapy sessions over a few days (e.g., 5 sessions over week), or few weeks of therapy (e.g., 4 weeks of therapy which is typical for clinical trials). This is equivalent to running a forward pass of Equation 1 over the training epoch T with fixed intervention variables $u(t)=u_{0}$ (e.g., fixed total dose, task, robotic assistance, etc.), as follows in Equation 3,

$$\begin{array}{cc} x\left(t_{0}+T\right) & =x\left(t_{0}\right)+\int_{t_{0}}^{t_{0}+T}f\left(x\left(\tau \right),u_{0};p\right)d\tau \\ y\left(t_{0}+T\right) & =g\left(x\left(t_{0}+T\right);p\right)+\epsilon \end{array}$$ (3)

where, $t_{0}$ is the start time of the therapeutic intervention, and $y(t_{0}+T)$ is the measured outcomes at the end of therapy at time $t_{0}+T$. Note that the training epoch must be fixed and clearly specified for the RAT-SCM to learn its parameters from data, and to correctly interpret and use it.

Although, proposed as a general causal model of RAT, at least some of the specific model parameters of the RAT-SCM will differ between robots. The model parameters of the RAT-SCM fall into two categories: (a) the set of values assumed by the nodes, and (b) the functional forms and parameters associated with the directed edges between the nodes. These model parameters will differ between robots based on at least one of the following factors, which include the tasks supported by the robot, the nature of the robotic assistance implemented in the robot, and the nature and types of external feedback provided during training.

### 3.3 Components of the SCM

“Everyone knows what a curve is, until he has studied enough mathematics to become confused through the countless number of possible exceptions.”

—Felix Klein

The nodes of the DAG in Figure 1 are some of the key variables/factors in the RAT-driven recovery process that influence the treatment outcome. The choice of these factors is based on the current literature and our understanding of sensorimotor recovery process. In the following subsections, we will provide definitions for each of these variables, to the extent currently possible. These definitions will focus on the nature of these variables, and more importantly, how these can be quantified and measured. Some of these definitions should be treated as working definitions, subject to refinement as our understanding of RAT matures. For each of these nodes we will also describe their known or hypothesized causes and effects as represented by the directed edges in the DAG. We will represent the variables corresponding to these nodes as $X_{i}$, where i will mostly be an uppercase alphabet of the node’s name (e.g., the task node will be $X_{T}$, task difficulty will be $X_{TD}$); it may include a lowercase alphabet when two nodes have names starting with the same alphabet (e.g., the $X_{Ch}$ is chosen for challenge to distinguish it from chronicity $X_{C}$). We will use the symbol ${Pa}_{i}$ to represent the set of parent nodes of $X_{i}$ (nodes that have a direct arrow pointing to $X_{i}$), and we will use $F_{i}(∙)$ to represent the function representing the causal relationship between $X_{i}$ and its parents ${Pa}_{i}$. Finally, we will use $U_{i}$ to represent the exogenous noise (52) term associated with $X_{i}$, which captures the unmeasured causes of $X_{i}$ and the random variability in $X_{i}$ that is not explained by its parents ${Pa}_{i}$.

#### 3.3.1 Task

The type of movements trained, along the joints and limb segments involved, influence the nature and amount of sensorimotor outcomes. The task node captures the information about the movements prescribed for training as part of RAT during the training epoch.

Definition 3.1 **Task**

*Task* $X_{T}$ is the specific movement types, involving a specific set of joints/limb segments, that are supported by a robot and are prescribed to a patient during the training epoch.

##### 3.3.1.1 Causes and effects

As a root node of the DAG (Figure 1), task has no parents $\left({Pa}_{T}=\emptyset \right)$. It defines the values assumed by the other four ingredient nodes:*Task Difficulty*, *External Feedback*, *Robotic Assistance*, and *Total Dose Prescribed*.

The task $X_{T}$ is a robot-specific node taking on values on a nominal scale consisting of the set of distinct movement-types supported by the given robot. We refer to this as the *task set* T,

$$T=\left\{t_{i}\;|\;i=1,\ldots ,n_{T}\right\}$$

where $t_{i}$ is the $i^{th}$ movement-type supported by the robot and $n_{T}=|T|$ is the total number of different movement types; both these are robot-dependent. For example, MIT-MANUS (32)—an end-effector robot—training only discrete point-to-point multi-joint arm reaching movements will have $n_{T}=1$. There is no canonical approach to identify and categorize distinct movement-types supported by a robot. Thus, the classification of distinct movement-types may not always be clear. For instance, the BONES (64, 65)—an exoskeleton robot—can train multi-joint (shoulder and elbow) and single joint (shoulder or elbow) discrete point-to-point reaching movements. Thus, in this case $n_{T}=3$ would be a reasonable choice. However, one could argue that $n_{T}=1$ for the BONES robot because multi-joint and individual joint training have similar therapeutic effects (66) and thus, from the recovery perspective, they need not be considered as distinct movement-types. A safer approach might be to use anatomical basis for task classification, while accounting for their similar therapeutic effects through the downstream causal links and their parameters. However, the most appropraite basis for task classification remains an unsolved problem in the field.

For a robot with task set T supporting $n_{T}>1$ tasks, a subject might be prescribed to train a subset of these tasks during the training epoch. The value of the task node could be represented by binary $n_{T}$-vector $X_{T}=[X_{T,1}\;X_{T,2}\;\cdots \;X_{T,n_{T}}{]}^{⊤}\in B^{n_{T}}$, where the $j^{th}$ element $X_{T,j}$ indicates if specific task $t_{j}\in T$ is prescribed. For instance, PLUTO (4)—a modular, multifunctional hand robot—can train discrete point-to-point movements involving the wrist flexion/extension ($t_{1}$), wrist ulnar/radial deviation ($t_{2}$), forearm pronation/supination ($t_{3}$), and hand opening/closing ($t_{4}$); thus, $n_{T}=4$. If a subject is prescribed to train two movement types, namely, wrist flexion/extension and hand opening/closing, then the task node would be presented as $X_{T}=[1\;0\;0\;1{]}^{⊤}$.

#### 3.3.2 Task difficulty

Task difficulty could be thought of as the information processing and execution demands required to perform a prescribed task/movement. Guadagnoli and Lee (84) divide task difficulty into two broad categories: *nominal* and *functional* task difficult. *Nominal difficulty* is the inherent demands of a task, independent of the performer and the environment. In the context of RAT-SCM, we define task difficulty as a restricted form of nominal difficulty of tasks performed during RAT without any physical assistance and augmented feedback.

Definition 3.2 **Task Difficulty**

*Task difficulty* $X_{TD}$ is the nominal difficulty associated with a task performed with the robot without any physical assistance or augmented feedback.

##### 3.3.2.1 Causes and effects

Task difficulty is determined by its single parent—task ${Pa}_{TD}=\{X_{T}\}$, and directly influences the *Challenge* node in the DAG.

Each task $t_{i}\in T$ has a set of parameters defining its spatiotemporal constraints, which determine its task difficulty. Task difficulty can be captured by a non-negative real number $R_{+}$, where increasing values indicate increasing task difficulty. For instance, a planar point-to-point discrete reaching movement task $t_{i}$ has several parameters, including, the total number of targets, their spatiotemporal locations, the order of target appearance, etc. The task difficulty of $t_{i}$ is determined by these parameter values. For instance, targets located closer to the subject would have lower task difficulty than farther targets, targets appearing a fixed order would have lower difficulty than the ones appearing in a random order.

For a given task set T and task prescription $X_{T}\in B^{n_{T}}$ (see Section 3.3.1), the task difficulty vector $X_{TD}=[X_{TD,1}\;X_{TD,2}\;\cdots \;X_{TD,n_{T}}{]}^{⊤}\in R_{+}^{n_{T}}$, where $X_{TD,j}$ is the task difficulty of task $t_{j}\in T$, defined as,

$$X_{TD}\leftarrow F_{TD}\left({Pa}_{TD},U_{TD}\right),\;X_{TD,j}=X_{T,j}\cdot f_{TD,j}\left(\pi_{j}\right)$$ (4)

where, $X_{T,j}$ indicates if $t_{j}$ is prescribed, $\pi_{j}\in R^{m_{j}}$ are the parameters defining the spatiotemporal constraints of $t_{j}$, $m_{j}\in Z_{+}$ is the number of parameters for the task $t_{j}$, and $f_{TD,j}\;:\;R^{m_{j}}\to R_{+}$ is the function that maps the task parameters of $t_{j}$ to its task difficulty. Note, that in Equation 4 task difficulty is zero when a task is not prescribed.

#### 3.3.3 External feedback

Feedback is essential for motor learning and recovery (85), playing both informational and motivational roles in the process (86, 87). The *external feedback* node refers to any additional, augmented, or enriched feedback provided to the subject during RAT. This includes, (a) visual and auditory feedback through gaming, virtual or augmented reality, (b) haptic feedback through the robot or external tendon vibration (78), (c) neuromuscular electrical stimulation (NMES) of motor and sensory structures (77), and (d) social, verbal or non-verbal feedback from a therapist or a caregiver interacting with the subject during therapy. Note that this feedback is over and above the intrinsic feedback (e.g., proprioceptive, tactile, visual etc.) naturally available to the subject during movement execution (86).

Definition 3.3 **External Feedback**

*External feedback* $X_{EF}$ is any augmented task-related feedback provided to the subject during RAT. This is information that is not naturally available to the subject during movement execution.

##### 3.3.3.1 Causes and effects

External feedback is determined by its single parent—task $\left({Pa}_{EF}=\{X_{T}\}\right)$, and directly influences three other nodes in the DAG: *Challenge*, *Motivation*, and *Sensory Afference*.

There is no canonical way to quantify and faithfully represent the multidimensional construct of external feedback. A rudimentary approach is to use a binary variable indicating the presence or absence of any augmented feedback during RAT. However, such a non-specific approach will club multiple dimensions of the feedback into a single variable, thus increasing response heterogeneity and masking causal effects. A more nuanced approach is to provide a detailed specification of the external feedback provided during RAT. Feedback could be decomposed into its distinct components and then quantified based on the type and amount of each of these components. These distinct components could include different sensory modalities (e.g., visual, auditory, or haptic) (88), feedback schedules (e.g., concurrent, terminal, faded, bandwidth etc.) (86), feedback content (e.g., knowledge of results, knowledge of performance etc.) (86), and social feedback (e.g., verbal praise, encouragement, etc.) (89). This multidimensional information about the external feedback for a prescribed task $t_{j}$ can be represented as a vector $\phi_{j}\in R^{n_{F}}$. The components of the vector $\phi_{j}$ could indicate the amount of the different distinct feedback components in the external feedback provided during training for the task $t_{j}$; $n_{F}$ is the number of parameters used to capture the details of this external feedback. The overall external feedback provided for the full set of tasks could then be represented by a matrix of parameters $\Phi \in R^{n_{T}\times n_{F}}$, where the rows corresponding to the prescribed tasks could have non-zero parameter values, while the rows corresponding to the non-prescribed tasks would be zero $0^{⊤}\in R^{n_{F}}$.

#### 3.3.4 Robotic assistance

The most characteristic feature of RAT is the physical human-robot interaction during therapy, which can take different forms (assistive, resistive, counterbalance, error-augmentation, etc.) (90). In the RAT-SCM, we will restrict our attention to the assistive form of physical interaction, where the robot applies external forces/torques on the human limb to augment voluntary movements. The assistance from the robot allows the subject to perform movements not possible voluntarily or perform them with reduced voluntary effort from the human neuromuscular system. Any physical interaction will qualify as robotic assistance only if the direction of the interaction forces/torques is: (a) aligned with the direction of the desired movement, i.e., $f^{⊤}v_{d}>0$, where f is the generalized force vector and $v_{d}$ is the generalized velocity vector in the direction of the desired movement; or (b) opposing gravity or the elastic forces from the limb’s antagonistic muscles to maintain the limb in a position, i.e., $-f^{⊤}f_{r}>0$, where $f_{r}$ is the restorative force due to gravity or antagonistic muscles. Together, these two conditions allow assistive interactions, along with arm deweighting and anti-gravity support, to be captured under the robotic assistance node. The *robotic assistance* node captures the amount of this form of physical interaction provided during RAT.

Definition 3.4 **Robotic Assistance**

*Robotic assistance* is the physical human-robot interaction during RAT that augments voluntary movements by enabling movements not possible voluntarily or to perform them with reduced voluntary effort.

##### 3.3.4.1 Causes and effects

Robotic assistance is determined by its single parent - task $\left({Pa}_{RA}=\{X_{T}\}\right)$, and directly influences three other nodes in the DAG: *Challenge*, *Motivation*, and *Sensory Afference*.

We can capture robotic assistance for a task $t_{j}$ as a non-negative real number $R_{+}$. The amount of robotic assistance provided during therapy depends on three factors: (a) the task to be completed, (b) the magnitude of force/torque applied by the robot, and (c) the subject’s physical effort in completing the task. When a subject is performing a task $t_{j}$ with robotic assistance, the resulting movement x(t) is generated by the combined effect of the subject’s active effort h(t) and the robotic forces/torques f(t). With the knowledge of h and f, we can define the amount of robotic assistance as the following,

$$a=\frac{\left\|f\left(t\right)\right\|}{\left\|h\left(t\right)+f\left(t\right)\right\|}\in R_{+}$$ (5)

It is assumed that, in Equation 5, voluntary effort h(t) is also expressed as forces or torques. According the above definition, (a) a=0, when the task is completed with no robotic assistance f(t)=0, and (b) a=1 when the task is completed with no active voluntary effort from the subject h(t)=0. Note that Equation 5, the denominator is the overall “input” that produces the movement “output” x(t). In principle, the denominator could be computed through an inverse model of the human-robot dynamics, as follow (Equation 6),

$$h\left(t\right)+f\left(t\right)=H^{-1}\left(x\left(t\right);t_{i}\right)\;⟹\;a=\frac{\left\|f\left(t\right)\right\|}{\left\|H^{-1}\left(x\left(t\right);t_{i}\right)\right\|}$$ (6)

One such approach using the cross-correlation between actual and simulated trajectories, based on an estimated dynamic model of the human arm, is used as a measure of “slacking” (56), which provides an indirect measure of the amount of assistance.

The overall robotic assistance provided for the full set of tasks could be represented by a vector $X_{RA}=[X_{RA,1}\;X_{RA,2}\;\cdots \;X_{RA,n_{T}}{]}^{⊤}\in R_{+}^{n_{T}}$, where the elements corresponding to the non-prescribed tasks would be zero.

#### 3.3.5 Total dose prescribed

*Total dose prescribed* refers to the total amount of therapy prescribed to the subject during the training epoch. Goikoetxea-Sotelo and van Hedel (91) define “total dose” as the product of dose and dosage, two multidimensional constructs defined similarly to their definitions in pharmacological interventions (91). *Dosage* consists of therapy session frequency and the total duration of therapy (91), while *dose* consists of therapy intensity and the duration of an intervention session (91). *Intensity* is defined as the amount of physical or mental effort put forth by a subject during therapy (91), which is not easy to quantify (91).

Definition 3.5 **Total Dose Prescribed**

*Total dose prescribed* $X_{TDP}$ is the total active therapy time prescribed to the subject during the training epoch for the prescribed tasks.

##### 3.3.5.1 Causes and effects

Total dose prescribed is determined by its single parent—task $\left({Pa}_{TDP})=\{X_{T}\right\}$, and directly influences two nodes in the DAG: *Motor Efference*, and *Sensory Afference*.

In the definition for “total dose prescribed”, we implicitly assume that the patient is 100% actively engaged in therapy. However, in practice the actual total dose received by a subject will be different from this value, depending on the actual training duration, the challenge of the tasks/movements being performed, and his/her level of engagement during therapy. This actual total dose will have a motor and sensory component, which are captured by the *Motor Efference* and *Sensory Afference* nodes in the DAG (see Sections 3.3.11 and 3.3.12, respectively). Ideally, $X_{TDP}$ should be represented by a vector of parameters capturing the different dimensions of dose and dosage, as defined by Goikoetxea-Sotelo and van Hedel (91). However, we restrict it to a single non-negative real number $R_{+}$ indicating the total active therapy time prescribed during the training epoch. This simplification is based on the hypothesis that the total active therapy time is the most important dimension influencing recovery (92). Note that although different frameworks exist for articulating dose in neurorehabilitation (91, 93), all of them are likely to collapse to the same scalar $X_{TDP}$, differing only in how they structurally decompose it.

#### 3.3.6 Motor impairment

According to the International Classification of Functioning, Disability and Health (94), an *impairment* is defined as a problem in the body structure or function such as a deviation or a loss. *Motor impairment* refers to impairments to the neuromuscular system impacting one’s ability to move.

Definition 3.6 **Motor Impairment**

*Motor impairment* $X_{MI}$ is the loss or abnormality of the neuromuscular anatomy or physiology that impacts one’s ability to produce skilled motor actions.

##### 3.3.6.1 Causes and effects

Motor impairment is a root node in the DAG, and thus has no parents $\left({Pa}_{MI}=\emptyset \right)$. It directly influences two other nodes in the DAG: *Challenge*, and *Motor Efference*.

Motor impairment captures several low-level motor control parameters, which have been traditionally captured through standardized clinical scales (e.g., Fugl-Meyer Assessment (57) for stroke), and more recently through sensor-based measurements (58, 95, 96). A generalized approach to capture motor impairments in the RAT-SCM is to represent it as a vector $X_{MI}\in R^{n_{M}}$. Here, the individual elements of $X_{MI}$ will represent different motor impairment parameters relevant to the specific clinical population being treated and the nature of the robot. For example, these could be the individual components of the FMA, joint range of motion, joint strength, movement quality measures, etc. The exact choice of the components of $X_{MI}$ for the RAT-SCM will depend on various interrelated factors: the nature of the robot, the limb segments and the motor control parameters being targeted, and the clinical condition being treated.

#### 3.3.7 Sensory impairment

The sensory impairment node is the sensory equivalent of the motor impairment node, capturing anatomical and physiological damage to the sensory structures relevant for motor control and learning. Sensory impairments are considered to be an important factor influencing motor recovery (59).

Definition 3.7 **Sensory Impairment**

*Sensory impairment* $X_{SI}$ is the loss or abnormality of the sensory anatomy or physiology that impacts one’s ability to produce and learn skilled motor actions.

##### 3.3.7.1 Causes and effects

Sensory impairment is a root node in the DAG, and thus has no parents $\left({Pa}_{SI}=\emptyset \right)$. It directly influences two other nodes in the DAG: *Challenge*, and *Sensory Afference*.

Similar to motor impairment, sensory impairment is also multidimensional and several standardized clinical scales (59), and technology-assisted assessments exist (60, 95, 97, 98). Sensory impairment can also be captured through a vector $X_{SI}\in R^{n_{S}}$; the exact scale choice depends on various interrelated factors.

#### 3.3.8 Chronicity

The chronicity node is the measure of the time since the neurological injury, which has a strong bearing on sensorimotor recovery in conditions like stroke (81, 83, 99).

Definition 3.8 **Chronicity**

*Chronicity* $X_{C}$ is the duration since the onset of the neurological injury.

##### 3.3.8.1 Causes and effects

Chronicity is a root node in the DAG, and thus has no parents $\left({Pa}_{C}=\emptyset \right)$. It directly influences *Sensorimotor Improvement* node in the RAT-SCM.

There is strong evidence from rodent studies that a transient critical sensitive period exists in the first few days/week following a stroke (99, 100), which resembles the period of heightened neuroplasticity in early development. Biernaskie et al. (100) showed that motor training in rodents interacts with this critical sensitive period to produce greater recovery than the same training provided outside this period. Zeiler et al. (101) showed that intense motor training in rodents after a second ischemic lesion led to an almost complete reduction in forelimb impairments left from the first lesion, demonstrating the strong influence of the critical sensitive period on moderating the impact of motor training on recovery. The phase II randomized, controlled trial—Critical Periods After Stroke Study (CPASS)—provided evidence for the existence of a similar critical sensitive period, where an additional 20 hrs of motor training along with usual care in the subacute phase led to the largest UL improvements (99).

The causal link from chronicity to sensorimotor improvement in the RAT-SCM captures the following effects of the critical sensitive period: (a) spontaneous recovery, which happens in the first few weeks and months after stroke. This influence decays over time and more importantly it is independent of any therapeutic intervention (82, 83), and (b) the synergistic interaction between the critical sensitive period and motor training that leads to greater recovery for the same training provided in the subacute phase (81, 99–101). The former effect is a direct causal effect that can lead to a change in sensorimotor improvements, the latter is a moderating effect that can change sensorimotor improvement only when training is provided.

Chronicity can be represented as a non-negative real number $R_{+}$, since this is a measure of time since the neurological injury. For instance, in stroke, chronicity can be measured in days since the stroke onset, which could be used as is in the final structural equation for sensorimotor improvements, or could be quantized to discrete levels (e.g., acute, subacute, chronic) before it is employed for modeling the influence of chronicity on sensorimotor improvements.

#### 3.3.9 Challenge

*Challenge* is believed to be a key driver of motor learning and recovery (84, 102–104). Although Guadagnoli and Lee (84) do not provide an operational definition of challenge, their arguments suggest that challenge is effectively functional task difficulty—the relative difficulty of a task with respect to the skill level of the performer and the environmental conditions (84).

Definition 3.9 **Challenge**

*Challenge* $X_{Ch}$ is the relative functional difficulty of a task with respect to the skill level of the performer and the environmental conditions.

##### 3.3.9.1 Causes and effects

Challenge is directly determined by five parent nodes in the DAG: *Task Difficulty*, *External Feedback*, *Robotic Assistance*, *Motor Impairment* and *Sensory Impairment* $\left({Pa}_{Ch}=\{X_{TD},X_{RA},X_{EF},X_{MI},X_{SI}\}\right)$. It directly influences two other nodes in the DAG: *Motivation*, and *Sensorimotor Improvement*.

Challenge or functional task difficulty shapes two important aspects of training: (a) the task-level “interpretable information” essential for the motor learning (84), and (b) the mental experience of the performer during training, involving mental workload, attention, motivation, etc. (103). The challenge node in the DAG is a parsimonious representation of these two aspects of training.

There are five causal arrows converging onto the challenge node in the proposed RAT-SCM. The nature of these causal influences are as follows: (i) Task Difficulty ($ch_{1}$): higher nominal difficulty increases challenge (84), (ii) Robotic Assistance ($ch_{2}$): assistance can decrease challenge for a given task, (iii) External Feedback ($ch_{3}$): appropriate feedback can decrease challenge, (iv) Sensory Impairment ($ch_{4}$) and Motor Impairment ($ch_{5}$): greater impairment increases challenge. Note that these relationships are likely to be non-linear and some non-monotonic.

Challenge is a latent construct and thus is not directly measurable; in Table 1 challenge is equated to an internal state variable in the general recovery model. However, there are several potential indirect measures of challenge. The interpretable information aspect of challenge can be measured through performance variables such a success rate, task error, movement variability, etc.; the exact choice of the performance measure will depend on the task being trained. The mental experience aspect can be measured through subjective questionnaires such as the NASA Task Load Index (61), which measures the mental workload experienced by a subject performing a task. More objective measures of mental experience include the enzymatic activity of the salivary α-amylase (105)—a biomarker of the activity of the sympathetic nervous system, reaction time during dual-tasking (105), electroencephalography (EEG)-based measures (68), etc. Challenge can be represented as a non-negative real number $R_{+}$. Thus, the challenge node in the RAT-SCM could be represented as a vector $X_{Ch}\in R_{+}^{n_{T}}$, a single number for each of the tasks.

$$X_{Ch}\leftarrow F_{Ch}\left({Pa}_{Ch},U_{Ch}\right),\;X_{Ch,j}=f_{Ch,j}\left(X_{TD,j},X_{RA,j},X_{EF,j},X_{MI},X_{SI},U_{Ch,j}\right)$$ (7)

where, $f_{Ch,j}$ is the function that computes the challenge for a task $t_{j}$ given the value of the variables in ${Pa}_{Ch}$ in Equation 7. The function $f_{Ch,j}$ will assign $X_{Ch,j}=0$ when $t_{j}$ is not prescribed, i.e., $X_{TD,j}=0$ (see Section 3.3.2).

#### 3.3.10 Motivation

The OPTIMAL theory (89, 106) proposed by Wulf and Lewthwaite emphasizes the importance of motivation in motor performance and learning. Motivation and its associated factors are believed to influence motor learning processes (107), and its role in patient outcomes are also well supported in the neurorehabilitation literature (62, 72, 89, 108). Motivation for neurorehabilitation is a highly dynamic (62, 72) and complex construct (109) influenced by: (i) factors intrinsic to the subject (e.g., personality, psychological status, depression, anxiety etc.), (ii) extrinsic factors (e.g., family and social support), (iii) disease severity and cognitive deficits, and (iv) factors related to the nature and content of the neurorehabilitation intervention. The last factor is the most relevant in the context of the RAT-SCM.

Definition 3.10 **Motivation**

*Motivation* $X_{M}$ is an internal state of a subject that drives behavior towards the goal of attaining functional recovery through neurorehabilitation.

##### 3.3.10.1 Causes and effects

Motivation is directly determined by three parent nodes in the DAG: *Challenge*, *Robotic Assistance*, and *External Feedback* $\left({Pa}_{M}=\{X_{Ch},X_{RA},X_{EF}\}\right)$. It directly influences one node in the DAG: *Motor Efference*.

The self-determination theory proposed by Ryan and Deci (110) broadly differentiates motivation into two categories: intrinsic and extrinsic. Intrinsic motivation drives activities for their inherent satisfaction; it is more desirable (110) for improved therapy adherence. While, extrinsic motivation regulates activities through external rewards or punishments, and comes in several forms with some forms being more desirable than others (110). The choice of appropriate levels of challenge, optimal robotic assistance, and the right type and amount of external feedback can build competence, autonomy, and relatedness—the three basic psychological needs identified by the self determination theory (110) to be conducive for intrinsic motivation for therapy.

Motivation and challenge have been shown to have an inverted U-relationship (67–69) with low challenge leading to boredom and high challenge leading to anxiety and frustration; both these extremes are detrimental to motivation. Thus, optimal challenge not only contains the maximal interpretable information for motor learning (84), but also maximizes intrinsic motivation (103). Robotic assistance has been shown to increase motivation compared to unassisted training after controlling for success rate (33); this supports the direct causal link between robotic assistance and motivation. External feedback can influence motivation through several means: (a) augmented audiovisual feedback through gaming, virtual or augmented reality can enhance motivation (72) by making therapy enjoyable, and (b) appropriately designed/scheduled task-related feedback can enhance motivation through increase perception of competence and self-efficacy (70). For example, providing feedback after successful trials has been shown to increase motivation (71). A recent multi-center survey on motivational factors in rehabilitation, the positive achievement emotions and self-efficacy was identified to be the top motivational factors by both patients and clinicians (111).

Motivation is commonly measured through self-reported questionnaires, such as the intrinsic motivation inventory (33), stroke rehabilitation motivation scale (62), motivation for rehabilitation scale (112), etc. Administering such questionnaires is not feasible on a regular basis to dynamically track the motivation levels of patients during therapy. In such scenarios, a visual analogue scale or 0–10 numerical scale could be employed as a coarse measure of motivation. Motivation levels for specific tasks might also be inferred from task adherence during therapy, with the assumption that higher adherence is indicative of higher motivation for that task. Similar to challenge, motivation can also be represented as a vector in $X_{M}\in R^{n_{T}}$.

#### 3.3.11 Motor efference

Active voluntary participation (103, 113) and sufficient dose (81, 91) are two necessary, and arguably the most important, factors for motor learning and recovery. The motor efference node captures this key information in the RAT-SCM that has a direct impact on recovery.

Definition 3.11 **Motor Efference**

*Motor efference* $X_{ME}$ is the total physical and mental effort exerted by the patient during therapy over the training epoch.

##### 3.3.11.1 Causes and effects

Motor efference has three parent nodes: *Motor Impairment*, *Motivation*, and *Total Dose Prescribed* $\left({Pa}_{ME}=\{X_{MI},X_{M},X_{TDP}\}\right)$. It influences two nodes: *Sensory Afference*, and *Sensorimotor Improvement*.

The causal link $me_{1}$ from motivation to motor efference captures the intensity of training, which was defined by Goikoetxea-Sotelo and van Hedel (91) as the amount of physical or mental work exerted by the patient during movement training. Increasing levels of intrinsic motivation is likely to increase both the intensity as well as adherence to training (73–75). Attentional resources may act as a confounder in the recovery in subacute stroke (114); attention during therapy is likely to be positively influenced by motivation. Adherence or the total actual therapy duration will be influenced by the total dose prescribed, which is captured by the causal link $me_{2}$. Finally, the actual motor output produced during training will also be influenced by the motor ability of the subject. More severely impaired patients produce smaller, slower, and poorly coordinated movements compared to mildly impaired patients; this is captured by the $me_{3}$ causal link.

Motor efference can be represented as a vector $X_{ME}\in R_{+}^{n_{T}}$ consisting of the motor efference for the individual tasks.

$$X_{ME}={\left[\begin{array}{ccc} X_{ME,1} & \cdots & X_{ME,n_{T}} \end{array}\right]}^{⊤}\leftarrow F_{ME}\left({Pa}_{ME},U_{ME}\right)$$ (8)

where, in Equation 8, $X_{ME,j}$ is the motor efference for task $t_{j}$. The individual motor efferences for each task could be computed as the product of the individual causal effects of its three parents, as the following,

$$X_{ME,j}=f_{ME_{1}}\left(X_{M,j}\right)\cdot f_{ME_{2}}\left(X_{TDP,j}\right)\cdot f_{ME_{3}}\left(X_{MI}\right)$$ (9)

where, $f_{ME_{1}}(∙)$, $f_{ME_{2}}(∙)$, and $f_{ME_{3}}(∙)$ in Equation 9 capture the functional relationship of the causal effect of motivation, total dose prescribed, and motor impairment on motor efference, respectively.

The causal link $f_{ME_{2}}(∙)$ is probably the easiest to model. This could be the total time spent training task $t_{j}$ with the robot. If the task $t_{j}$ is not prescribed, this function returns 0, to indicate that $t_{j}$ was not trained, else it could return a non-negative number. The other two causal links are harder to model. There is currently no standard approach to measure the intensity of neurorehabilitation training Goikoetxea-Sotelo and van Hedel (91). A potentially useful behavioral correlate could be movement vigor—a heterogeneous construct related to the velocity or duration of a movement relative to its extent (115). Faster movements to a target indicate increased subjective value associated with the target (116), signaling heightened motivation. Faster movements require higher physical effort, and possibly higher mental effort to ensure accuracy to mitigate signal-dependent noise (117). However, such behavioral measures of intensity/effort will need to be normalized to account for the nature of a task, and subject-dependent factors, such as age, gender, weakness, etc. (91). Other physiological measurements that could be employed for this purpose include skin conductance (118), error-related potentials in EEG (119), salivary α-amylase activity (105), etc. However, not all such measurements may be practical for routine use.

#### 3.3.12 Sensory afference

Sensory feedback is vital for movement planning, execution, and learning (120). Any movement will generate sensory signals that ascend back to the central neural structures shaping movement performance and learning. This can involve any of the four sensory modalities: somatosensory, vision, auditory, and vestibular.

Definition 3.12 **Sensory Afference**

*Sensory Afference* $X_{SA}$ is the total quantity and quality of sensory feedback provided to the subject during therapy over the training epoch.

##### 3.3.12.1 Causes and effects

Sensory afference has five parent nodes: *Robotic Assistance*, *Sensory Impairment*, *External Feedback*, *Total Dose Prescribed*, and *Motor Efference* $\left({Pa}_{SA}=\{X_{ME},X_{SI},X_{EF},X_{RA},X_{TDP}\}\right)$. It influences one node: *Sensorimotor Improvement*.

The quantity of sensory feedback refers to the cumulative magnitude of sensory stimulation (e.g., area under the sensory stimulation magnitude curve). Quality, on the other hand, refers to a measure of congruence or alignment between the intended task and the sensory feedback provided during task performance. Thus, sensory afference can be thought of as the total amount of “task-aligned” sensory feedback provided during therapy over the training epoch. We can represent sensory afference as a vector $X_{SA}=[X_{SA,1}\;X_{SA,2}\;\cdots \;X_{SA,n_{T}}{]}^{⊤}\in R^{n_{T}}$ of real numbers; unlike motor efference we could allow sensory afference to assume negative values to indicate anti-correlated sensory feedback. The sensory afference $X_{SA,j}$ for a given task $t_{j}$ could be computed as the following over the training epoch T,

$$X_{SA,j}=f_{SA_{3}}\left(X_{SI}\right)\cdot \left(\int_{T}{\hat{\phi }}_{\;j}{\left(t\right)}^{⊤}W\phi_{\;j}\left(t\right)dt\right)$$ (10)

where, $f_{SA_{3}}(∙)$ captures the “filtering” effect of sensory impairment on the external feedback in Equation 10. The second term computes the total “task-aligned” sensory feedback while training task $t_{j}$ over the training epoch. ${\hat{\phi }}_{\;j}(t),\phi_{\;j}(t)\in R^{n_{F}}$ are vector functions of time over the interval (0,T) representing the expected and actual sensory feedback for the task $t_{j}$. W is a positive definite matrix representing the weights for the different sensory feedback components.

Sensory feedback during training can be generated and moderated by various factors. The causal link $sa_{1}$ represents the sensory feedback generated by the voluntary movement performed by the subject. Robotic assistance can produce larger and faster movements than that produced by pure volition, and can thus result in augmented sensory feedback ($sa_{2}$). A robot applies external forces on the limb segment, which activates the spindle and Golgi tendon afferents of the antagonistic muscles, along with other joint afferents and some somatosensory afferents at the point of application of the force. A wide range of sensory feedback can be provided through the causal link $sa_{4}$ from the external feedback node. These could include, NMES which can activate a range of sensory and motor structures (77) beyond what is possible with robotic assistance, muscle tendon vibration (78), augmented visual or auditory feedback (76), etc. The causal link $sa_{5}$ will play a role in determining the actual dose of sensory feedback provided during therapy, similar to its influence on motor efference $\left(me_{2}\right)$. The effect of these four causal links $\left(sa_{1},sa_{2},sa_{4},sa_{5}\right)$ could be represented by the integral term in Equation 10. The quantity and quality of this sensory feedback will be moderated by the subject’s level of sensory impairment, indicated by the causal link $sa_{3}$; this is represented by $f_{SA3}(∙)$ in Equation 10. This is similar to the moderating effect of motor impairments on motor afference $\left(me_{3}\right)$.

#### 3.3.13 Sensorimotor improvement

The sensorimotor improvement node is the outcome or the target node of the RAT-SCM that captures the change in the subject’s sensorimotor behavior undergoing RAT. In their paper on the rehabilitation treatment specification system, Hart et al. (23) proposed that the targets must be specific proximal changes expected from a treatment. Thus, given the nature of RAT, the sensorimotor improvement node will be restricted to measurements of impairment and low-level motor control variables.

Definition 3.13 **Sensorimotor Improvement**

*Sensorimotor Improvement* $X_{SMI}$ is the change in low-level motor control and impairment level variables of a subject undergoing RAT over the given training epoch.

##### 3.3.13.1 Causes and effects

Sensorimotor improvement has four parent nodes: *Motor Efference*, *Sensory Afference*, *Challenge*, and *Chronicity* $\left({Pa}_{SMI}=\{X_{Ch},X_{ME},X_{SA},X_{C}\}\right)$.

RAT can lead to improvements in various impairment-level and motor control variables. These include improvements in joint range of motion(121, 122), movement speed (63, 123), reaching accuracy (63), movement smoothness (63, 123), path efficiency of arm reaching movements (63), grip strength (121), etc. These are usually measured using sensor-based systems or robots. In addition to this, there is also good evidence supporting the improvement of UL impairments after RAT in stroke as measured by the FMA (8). The sensorimotor improvement node captures the change in these variables over the training epoch, which can be represented as a vector $X_{SMI}\in R^{n_{O}}$, where $n_{O}$ is the number of impairment/motor control variables targeted by the robot. Note that these impairment measurements can include joints not directly trained during therapy, to account for the generalization of therapeutic gain to the proximal UL when training the distal UL (124).

The combined effect of the four causal links $smi_{1},smi_{2},smi_{3}$, and $smi_{4}$ on the sensorimotor improvement node can be represented as,

$$X_{SMI}\leftarrow F_{SMI}\left({Pa}_{SMI},U_{SMI}\right)$$ (11)

where, in Equation 11, $F_{SMI}$ represents the causal mechanism determining $X_{SMI}$ given ${Pa}_{SMI}$. The function $F_{SMI}$ will be a highly complex, non-linear map that captures the multitude of motor learning processes at play during therapy (49, 84, 103, 125). The motor efference $X_{ME}$ and sensory afference $X_{SA}$ nodes drive these processes stimulating the central neuroplastic (49, 107), and peripheral neuromuscular/soft tissue changes (80) that underlie sensorimotor improvements. The causal effects $X_{ME}$ and $X_{SA}$ on the central neuroplastic changes are likely to be moderated by internal brain states, such as experienced challenge (61, 84, 103) and motivation (89, 103, 126, 127). This is captured by the causal link $smi_{3}$. The effects of chronicity (81–83, 99–101) on sensorimotor improvement—both a direct effect (spontaneous recovery, independent of training) and a moderating effect on the impact of the training-related inputs—are captured by $smi_{4}$ (see Section 3.3.8).

## 4 Implications of the RAT-SCM

“Prediction is very difficult, especially about the future.”

—Niels Bohr

**Figure 2:**
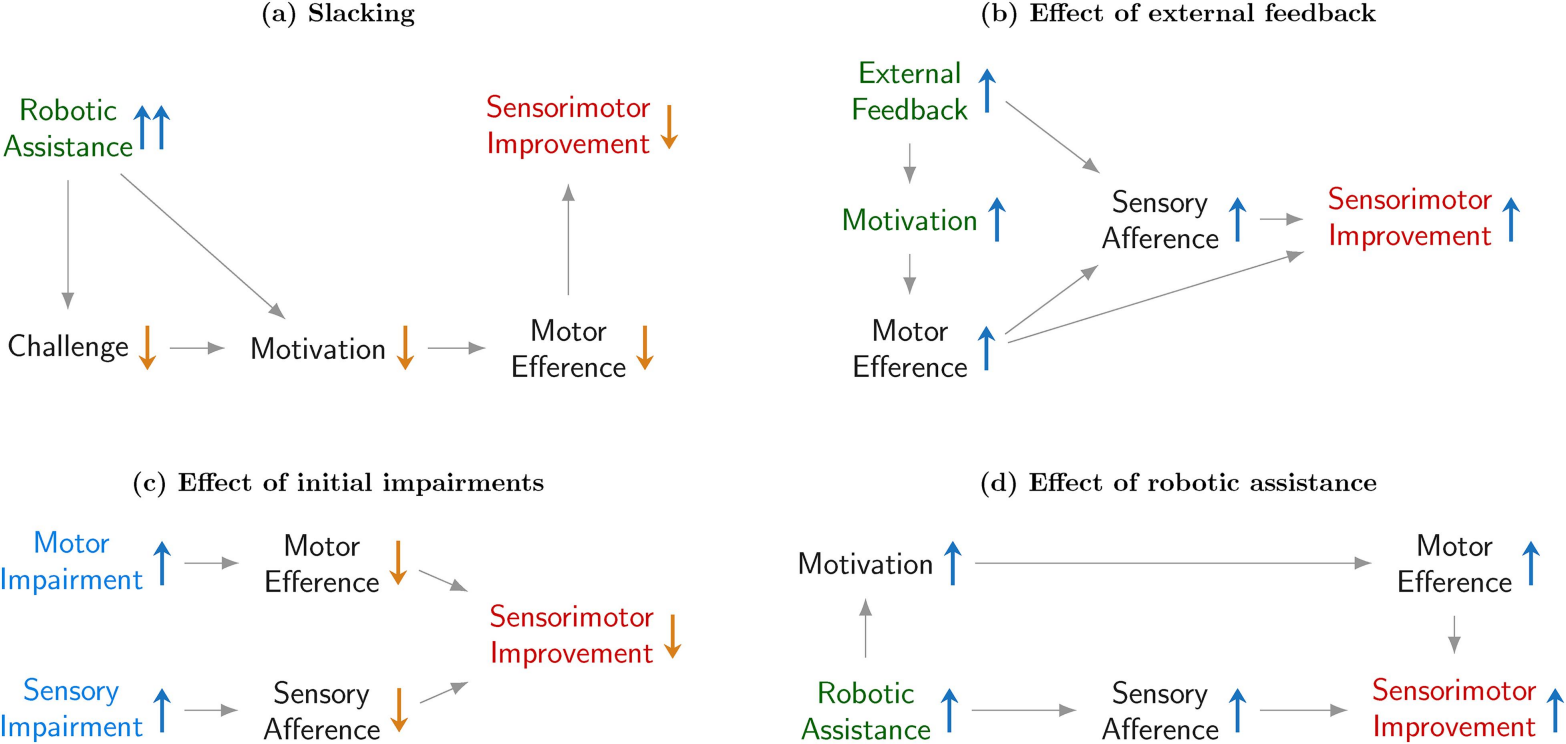
Subgraphs of the RAT-SCM depicting the causal reasoning behind some of the implications of the model discussed in the Section 4. The blue-colored up arrow and orange-colored down arrow represent the direction of change of a particular node. Double arrows represent a very large increase or decrease in the value of a node. The four subplots depict the sub-graph based explanation for the four implications of the RAT-SCM (a) slacking, (b) effect of external feedback, (c) effect of initial impairments, and (d) effect of robotic assistance.

The utility of any model is judged by the known phenomena it can explain and the testable predictions it makes about the world. In this section, we will highlight some of the phenomena associated with RAT explained by the proposed RAT-SCM, along with some predictions of the model, wherever applicable. Each of these phenomena and predictions can be explained by one or more causal routes in the RAT-SCM, which are depicted in Figure 2 as subgraphs.

### 4.1 Slacking in RAT

Slacking is a commonly observed phenomenon in RAT, where the human subject reduces his/her effort when there is more robotic assistance than required to complete a given task (128). In the RAT-SCM, high levels of robotic assistance can reduce challenge, which in turn reduces motivation, and thus motor efference (or effort). Casadio and Sanguineti (129) provide some support to the negative impact of slacking on the change in motor impairments, as measured using the FMA. Fitting a linear state space model to data from a RAT clinical study, they found that the estimated parameter for the slacking term was correlated to the change in the FMA score for the individual subjects; subjects displaying more slacking had smaller gains in motor impairment (129). In the extreme case, maximal slacking results in purely passive movements, that do not produce any appreciable improvements in motor function or impairments (130). In the RAT-SCM, passive movements would have zero challenge and motor efference (no voluntary effort), thus resulting in no appreciable sensorimotor improvement; changes to peripheral soft tissue might still happen (80).

Figure 2a depicts the causal pathway explaining slacking in RAT. A very high level of robotic assistance reduces challenge and motivation, which leads to reduced active involvement (motor efference), producing poor sensorimotor improvement. Note that there is evidence indicating that increased robotic assistance produces increased motivation (19, 33), which one could argue compensates for the reduced challenge. However, we hypothesize that motivation cannot monotonically increase with robotic assistance because the reduction in challenge will eventually lead to boredom and reduced motivation.

### 4.2 Effect of augmented sensory feedback

There is evidence to support the positive effects of augmented external feedback on the recovery of motor impairments of the UL post-stroke (131). A recent meta-analysis comparing the effect of BCI-triggered assisted training with robots or NMES found that BCI+NMES had significantly large improvements in motor function compared to BCI+Robots (132, 133). This could be attributed to the differences in the nature and amount of sensory feedback resulting from NMES compared to robots. NMES produces larger activation of the sensory afference node in congruence with motor efference generated through voluntary effort, thus resulting in larger sensorimotor improvement. Both robotic and NMES assistance activate the spindles and the Golgi tendon organs of the antagonists. However, NMES additionally activates the agonists, the cutaneous sensory structures below the electrodes, and other sensory nerves along the path of the electrical current. Another recent study employing tendon vibration in conjunction with RAT demonstrated larger benefits for the tendon vibration group than the one without (78). This too can be explained as the consequence of increased sensory afference in the RAT-SCM.

Figure 2b shows that an increase in external sensory feedback can have a positive effect on motor efference, through motivation, and sensory afference and thus on sensorimotor improvement. Note that the effect on sensorimotor improvements can be through the causal route involving motor efference for reward-based augmented feedback modalities targeting motivation. Other modalities, like tendon vibration, TENS (88), are more likely to have an effect primarily through the causal path involving sensory afference, without having an appreciable effect on motor efference.

### 4.3 Effect of initial impairments

The baseline impairment plays a moderating role on the amount of improvement following RAT (134, 135). Similar moderating effects have been identified for baseline sensory impairments on gains made from RAT (15, 16). These observations can be explained by two causal routes in RAT-SCM. Impairments have a moderating effect on the motor efference (Equation 9) and sensory afference (Equation 10) during therapy, which in turn impact sensorimotor impairment. The levels of impairments could also limit the levels of challenge and motivation achievable through RAT, and thus impact recovery. For instance, a severely impaired subject might find training challenging even with high levels of assistance.

This effect is depicted in Figure 2c where the baseline or initial impairment levels have a moderating effect on motor efference and sensory afference—the two vital nodes mediating the effect of the ingredient nodes on sensorimotor improvement. Increased impairment levels reduce motor efference and sensory afference, thus reducing sensorimotor improvements. Note that the effect of impairments on challenge and motivation is not shown in Figure 2c because the therapist planning the therapy will account for the impairment levels and appropriately set the other ingredient nodes to ensure optimal achievable challenge and motivation.

### 4.4 Effect of robotic assistance

The role of robotic assistance in sensorimotor recovery has been a topic of interest since the early days of RAT (136, 137). Surprisingly, only a handful of studies have investigated this issue while controlling for the relevant confounders. Our recent meta-analysis evaluating such studies found that robotic assistance has a significant effect (standardized mean difference ≈ 0.4) on motor impairments with similar effects on both arm and hand training (138). The small overall sample size (≈300) and moderate heterogeneity ($I^{2}\approx 70\%$) of these studies mean these results have to be treated with caution. However, this result warrants future systematic evaluation of the clinical value of robotic assistance.

The proposed RAT-SCM model predicts that the presence of robotic assistance will result in better outcomes than unassisted training. This causal effect on sensorimotor improvement can be through: (a) enhanced motivation leading to more training (139) and (b) enhanced sensory afference; note that the third potential route from challenge to sensorimotor improvement has a moderating effect on the other two routes. The motivational route of robotic assistance on recovery is known (15, 19, 33, 139). The other and arguably more interesting route is the one through enhanced sensory afference engaging Hebbian-like learning mechanisms (19, 140, 141). A recent study Farrens et al. (15) found that the standard RAT leads to better outcomes only in patients with intact proprioception, lending some support to the existence of this sensory afference-mediated causal route. Another interesting prediction of the RAT-SCM is that NMES-assisted therapy will lead to better outcomes than RAT, once we control for the task, task difficulty, external feedback, and total dose.

Figure 2d explains the role of robotic assistance and the prediction through the enhanced motivation from the choice of optimal robotic assistance, and the stronger sensory afference resulting from assisted movements. With NMES-assisted movements, the sensory afference is likely to be stronger for the same level of motor efference, which is the rationale for predicting better outcomes with NMES-assisted movements compared to RAT.

## 5 Limitations of the RAT-SCM

“The truth of the story lies in the details.”

—Paul Auster

The RAT-SCM is the first attempt at a comprehensive model for the mechanistic understanding of RAT. It brings together most of the known important factors involved in RAT and proposes a set of causal links between them, mostly based on the existing literature. The chosen level of abstraction for the model strikes a balance between the specificity of the underlying mechanistic processes and the practical aspects, such as measurability of its individual factors, identifiability of causal links, and the model’s clinical utility. The RAT-SCM, as currently specified, has important limitations that we make explicit in this section. A clear understanding of these limits is essential both for interpreting the model appropriately and for guiding future work aimed at refining and extending it.

### 5.1 Node definition and measurement

Many of the node definitions provided in the RAT-SCM are working definitions with little consensus from the field. Without agreed definitions, standardized measurement of these nodes will be difficult. It is wise to avoid premature definitions and avoid mistakes previously made in the field. For instance, movement smoothness—an important construct—was being quantified with inappropriate methods (142–144) for several years due to the lack of consensus on its definition and on what constituted a valid measure of smoothness. While striving to find an agreeable definition, a balance must be struck between the specificity of the construct and its routine measurability, preferably involving minimal manual processes.

### 5.2 Restrictions to assistive forms of robotic interaction

The proposed model is restricted to robotic assistance, while there are numerous other forms of physical human-robot interaction Marchal-Crespo and Reinkensmeyer (90). These include, error amplification (145, 146), active constrained assistance (147), resistive strategies (148), etc. The focus on robotic assistance was because this is the most common form of robotic interaction implemented in RAT. This restriction can be easily addressed by modifying the robotic assistance node through: (a) appropriate encoding and quantification of the different types of robotic interactions, and (b) modification of the functions corresponding to the causal links coming out of this node; for example, error amplification and resistance might enhance sensory afference beyond assisted training. The impact on motivation is also likely to be different for the different types of interactions.

### 5.3 Lack of training-recovery loops

DAGs are by definition acyclical (52). RAT is not a simple feedforward process; for instance a patient’s current impairment level influences his/her recovery, which in turn changes his/her impairment level in the future. One way to address this issue is to unfold the DAG over time (52). A different instance of the same DAG is considered for each training epoch, with causal arrows between consecutive training epochs. For example, the sensorimotor improvement at the time instant k will impact motor/sensory impairment at time k+1; this corresponds to a causal arrow from the sensorimotor improvement node from time k to the motor and sensory impairment nodes in next time k+1.

### 5.4 Lack of information about capacity to improve

Hardwick et al. (149) showed that stroke subjects with the same level of performance need not have the same capacity for improvement or learning. In particular, they found that the stroke subjects trained on a particular task are not the same as untrained ones with a lower impairment. Thus, impairment or performance levels alone are insufficient to determine improvement following an intervention. Its unclear how this information could be captured in the RAT-SCM. One possibility is to incorporate an additional node that captures the “learning reserve” or “learning capacity” of a subject. This node is likely to depend on the initial impairment after stroke (SAFE score or the presence of MEP) (20, 21), and the history of intervention provided to the subject. However, the exact nature of this node and its measurement currently remain unknown.

### 5.5 Others

In addition to the aforementioned limitations, there are other limitations worth noting. (a) The proposed RAT-SCM is not complete in capturing all the relevant factors and causal links. Its likely that there are other important factors not included in the DAG, along with missing or incorrectly specified causal links. For instance, the only two nodes capturing subject characteristics in the RAT-SCM are the motor and sensory impairments. Impairments are necessary but are most probably insufficient. A more realistic RAT-SCM will need to consider a detailed set of subject traits including, age, personality, fatigue, cognitive impairments, neurophysiological parameters, social/family support etc. The causal effects of these subject traits will need to be incorporated to improve the validity of the RAT-SCM. (b) The model, its discussion, and the supporting literature are primarily focused on the UL RAT in stroke. The applicability of the model to other conditions and to the lower-limb will need to be carefully investigated by experts from those areas.

## 6 Using the RAT-SCM in clinical practice

“In theory there is no difference between theory and practice, while in practice there is.”

—The Yale Literary Magazine, 1882

Although, the proposed SCM is formulated as a general model of RAT-driven sensorimotor recovery, its details will be robot specific. The RAT-SCM model will need to be instantiated for a given robot (e.g., MIT-MANUS, PLUTO) and its parameters identified through data obtained from observations and experimental studies. These learned parameters will include, the functional relationships corresponding to the various causal links, and the distribution of the exogenous variables associated with the different nodes. For the sake of this section, we will assume that we have an accurate RAT-SCM for a particular neurological condition, which has been fully specified for a given robot. This causal model can be used for implementing precision neurorehabilitation. Given a particular patient with some characteristics and health-related parameters p, along with a set of constraints and preferences c, in Equation 2, for RAT, we can use the causal model to answer two important RAT planning questions, in the following order:

- **Q1.** What is the maximum expected treatment outcome for the given patient over the training epoch?
- **Q2.** What are the optimal set of therapy parameters (values for the ingredient nodes) that results in this maximum expected treatment outcome?

First, we would pose **Q1** taking into consideration the patient characteristics and constraints, which is an optimization problem in Equation 12, over the RAT-SCM (150) searching over the space of parameter values of the ingredient nodes.

$$\begin{array}{cc} J^{*}=\underset{X\in X}{\text{maximize}} & \;J(X_{SMI},X;p)\\ \text{subject to} & \;X_{SMI}=G(X,p)\\ & \;c\left(X,p\right)\leq 0 \end{array}$$ (12)

where, $X=(X_{T},X_{TD},X_{RA},X_{EF},X_{TDP})$ is a tuple of the ingredient nodes. X is the set of all possible values for tuple of ingredient nodes X. The function $J(∙)\;:\;R^{n_{O}}\mapsto R$ computes the overall sensorimotor improvement from the $X_{SMI}$. G(∙) is the overall function capturing the causal mapping from the set of ingredient nodes and the patient characteristics to the sensorimotor impairment node. The constraints and preferences of the subject are captured by the vector function c(∙). The number $J^{*}$ is the maximum expected sensorimotor improvement for the given subject characteristics p and constraints c.

If $J^{*}$—the answer to **Q1**—is sufficiently high, then prescribing RAT for the given subject would be deemed worthwhile. At this stage, we would query the model for the optimal values of the ingredient nodes that produces $J^{*}$.

$$\begin{array}{cc} X^{*}=\underset{X\in X}{\arg\max} & \;J(X_{SMI},X;p)\\ \text{subject to} & \;X_{SMI}=G(X,p)\\ & \;c\left(X,p\right)\leq 0 \end{array}$$ (13)

where, in Equation 13, $X^{*}$ is the set of values for the ingredient nodes that can give us the maximum expected sensorimotor improvement $J^{*}$.

The above discussion is meant to only demonstrate how a causal model can be employed for rationally prescribing RAT for a given patient. The actual implementation of this process is contingent on the availability of a fully specified RAT-SCM model, and the availability of appropriate constrained optimization algorithms over the causal DAGs (150). Such a fully specified RAT-SCM—its structure and parameters—can be learned in practice from appropriately designed experimental and observational studies. This is the focus of the following section (Section 7).

## 7 Learning the RAT-SCM in practice

There are two aspects to learning a causal model: (a) learning the model structure, i.e., which causal edges exist in the model, and (b) learning the model parameters, i.e., the parameters of the functional forms associated with the individual causal edges; the two aspects are interrelated and inform each other. For instance, the absence of a significant parameter for a particular edge can be used to infer the absence of that edge in the model structure.

Inferring the model structure from data—the problem of *causal discovery*—is considerably harder and less settled than the parameter learning problem. Existing methods for causal discovery rely on constraints placed on conditional independencies (151), methods exploiting asymmetries introduced by causal edges in the data generating process (152), and score-based search of the graph space for the best-fitting structure (153). These methods rely on strong assumptions, are sensitive to sample size and measurement error, and typically only recover a Markov equivalence class of DAGs rather than a unique graph. Thus, causal discovery of the DAG for RAT must be employed as a complement to expert knowledge rather than as a replacement. The proposed RAT-SCM can serve as a prior, while its implied conditional independencies serve as falsifiable predictions that can prune and refine the model structure. Given that we currently have a tentative model structure for the RAT-SCM (Figure 1), we will focus on the learning of the model parameters associated with the individual causal edges of the RAT-SCM. This section is meant to be illustrative, rather than exhaustive. A full treatment of estimation and identifiability for the RAT-SCM is the subject of our forthcoming work.

Once a causal structure is known or assumed, identifying and estimating the associated interventional effects is a comparatively mature problem, supported by a well-developed body of methods spanning the graphical/structural tradition (52) and the potential-outcomes and design-based econometric traditions (26, 27, 154).

Causal models can be learned in a piecemeal manner, where each causal link can be learned individually through a combination of appropriately designed observational and experimental studies. A key practical benefit of an explicit causal graph is that it directly informs study design: for any target link, the graph identifies which variables must be adjusted for—and which must not—to obtain an unbiased estimate of a causal effect, and equivalently which to randomize or restrict by design. A few illustrative examples for the RAT-SCM are provided in Table 4, where some design details are provided for learning the parameters of three selected causal links in the RAT-SCM either through experimental or observational studies. These three links are deliberately chosen to illustrate three distinct identification situations: a link that can be estimated through randomization alone ($m_{1}$), a link requiring control of additional variables beyond randomization ($m_{2}$), and a link for which experimentation may be infeasible, so that the effect—and its moderation—must be estimated observationally ($me_{1}$).

**Table 4:** Study design for learning the parameters of selected causal links in the RAT-SCM.

Causal link of interest	Experimental study design	Observational study design
$m_{1}\;\;:\;X_{Ch}\to X_{M}$	**Randomize** Challenge.	**Adjust** for Robotic Assistance, External Feedback.
$m_{2}\;\;:\;X_{RA}\to X_{M}$	**Randomize** Robotic Assistance.	**Adjust** for Challenge, External Feedback.
	**Fix** Challenge, External Feedback.	
$me_{1}\;\;:\;X_{M}\to X_{ME}$	${}^{†}$**Randomize** Motivation.	**Adjust** for Total Dose Prescribed, Motor Impairment, and **model** their interaction with Motivation.
	**Model** the interaction of Total Dose Prescribed and Motor Impairment with Motivation.	

The first column lists the causal link of interest, while the second and third columns provide the experimental and observational study design, respectively, for learning the parameters of that link. Note that all nodes mentioned in the study columns need to be measured along with the parent and child node of each causal link of interest. The gray colored cell indicates that the proposed experimental study may not be feasible due to ethical or practical concerns. “†”: Downward manipulation of motivation may not be ethically permissible, while upward manipulation may suffer from ceiling effects; both make a controlled experiment difficult.

In Table 4, estimating $m_{1}$, the direct effect of *Challenge* on *Motivation*, requires randomizing *Challenge*. Since there are no other mediated paths to *Motivation* from *Challenge*, this link can be identified through simple randomization. For example, using success rate as a surrogate for *Challenge*, an experiment evaluating the effect of success rate on motivation can provide an estimate of this link. However, if we employed an observational study to estimate $m_{1}$, we would need to adjust for the backdoor paths through *Robotic Assistance* and *External Feedback*.

Estimating $m_{2}$, the direct effect of *Robotic Assistance* on *Motivation*, through an experimental study requires a slightly different approach. We need to randomize *Robotic Assistance* while fixing the other two parent nodes of *Motivation*—*Challenge* and *External Feedback*. Fixing *Challenge* is essential to close the mediated path from *Robotic Assistance* to *Motivation* through *Challenge*.^2^ Note that *Challenge* is a collider on the paths from *Robotic Assistance* and *External Feedback* to *Motivation*, so fixing *Challenge* will induce collider bias, which can be addressed by fixing *External Feedback*. If we were to use an observational study design, adjusting for *Challenge* blocks the indirect path from *Robotic Assistance* to *Motivation* through *Challenge*, thereby isolating the direct effect $m_{2}$, while adjusting for *External Feedback* both closes the backdoor path from *Robotic Assistance* to *Motivation* through *Task* and *External Feedback*, and corrects the collider bias induced by conditioning on *Challenge*.

The direct effect $me_{1}$ of *Motivation* on *Motor Efference* can be estimated through an experimental study by simply randomizing *Motivation*. However, such an experiment may be difficult to realize. Manipulating motivation downward—deliberately inducing low motivation for training—is ethically impermissible, while upward manipulation may suffer from ceiling effects in already-motivated patients. Thus, the $me_{1}$ link is best estimated through an observational design. There are several backdoor paths from *Motivation* to *Motor Efference*, all of which can be blocked by adjusting for *Motor Impairment* and *Total Dose Prescribed*. Additionally, in the observational study, the moderating effect of *Motor Impairment* and *Total Dose Prescribed* can be captured by modeling their interaction with *Motivation*.

Similar study designs can be formulated for estimating the parameters of the other causal links described in the RAT-SCM. As noted in Section 3.2, at least some of these parameters are likely to be robot-dependent, in particular the ones from the ingredient nodes. The ones between the latent nodes (corresponding to x in Table 1) are more likely to be robot-independent, given that these are related to the sensorimotor learning process associated with the brain. Across all these designs, valid and practical measurement of the latent constructs (e.g., *Challenge*, *Motivation*, and *Motor Efference*) remains a key prerequisite, and is itself a non-trivial challenge.

## 8 Conclusion

The proposed RAT-SCM is timely because device development has far outpaced mechanistic understanding of RAT for UL neurorehabilitation. The SCM pinpoints to specific key variables identified from the UL RAT literature, along with the known/hypothesized causal links between them. This explicit, concrete, critique-able causal architecture allows principled reasoning about interventional arguments (e.g., effect of robotic assistance, dose), confounding variables, and mediating and moderating mechanisms (e.g., impact of initial impairments), thus enabling scientific discourse beyond associational and heuristic arguments (e.g., biomarkers correlated with recovery; see Section 1).

Nevertheless, the proposed RAT-SCM is provisional and necessarily incomplete (see Section 5). It is a useful, structured synthesis of our current understanding of RAT, and not a complete, unquestionable description. The model’s components, its implications, and predicted conditional independencies must be challenged with evidence and consensus. First, carefully designed multi-disciplinary expert consensus studies will be necessary for accurate and useful construct definitions, along with valid and practical measurement methods. Second, future observational and experimental studies must take on the mantle of empirical falsification, refinement of the nodes and the causal edges, and the incorporation of the missing constructs (e.g., additional subject traits; see Section 5). As pointed out in the manuscript, this problem can be approached in a piecemeal manner by addressing selected sections of the DAG and its components.

The clinical planning demonstration illustrates how, in theory, a fully specified RAT-SCM for a given robot and indication can support precision neurorehabilitation for individual patients. The proposed model provides a principled way to frame questions about the attainable outcomes under a given set of constraints, along with the therapy parameters for this optimal outcome.

In conclusion, this work is not a claim to a definitive mechanistic account of RAT, but an effort to put forth, on the table, a tangible causal graph for RAT-driven recovery. The hope is that this causal model will set off a cascade of systematic critique, empirical testing, and iterative revision by the neurorehabilitation, robotic rehabilitation, and causal-inference communities, moving the field towards shared, testable causal models that can genuinely inform the design and prescription of robot-assisted therapy.

## Data Availability

The original contributions presented in the study are included in the article/Supplementary Material, further inquiries can be directed to the corresponding author/s.

## Footnotes

^1^Shoulder, elbow, wrist and/or hand.

^2^Rowe et al. (19) reported a causal effect of robotic assistance on motivation; however, this effect is not purely $m_{2}$, but a combination of the direct $m_{2}$ and indirect effects $ch_{2}-m_{1}$ mediated by *Challenge* (success rate).
